# Functional characterization of three trehalase genes regulating the chitin metabolism pathway in rice brown planthopper using RNA interference

**DOI:** 10.1038/srep27841

**Published:** 2016-06-22

**Authors:** Lina Zhao, Mengmeng Yang, Qida Shen, Xiaojun Liu, Zuokun Shi, Shigui Wang, Bin Tang

**Affiliations:** 1 Hangzhou Key Laboratory of Animal Adaptation and Evolution, College of Life and Environmental Sciences, Hangzhou Normal University, Hangzhou, Zhejiang, 310036, China

## Abstract

RNA interference (RNAi) is an effective gene-silencing tool, and double stranded RNA (dsRNA) is considered a powerful strategy for gene function studies in insects. In the present study, we aimed to investigate the function of trehalase (TRE) genes (*TRE 1-1*, *TRE 1-2*, and *TRE-2*) isolated from the brown planthopper *Nilaparvata lugens*, a typical piercing-sucking insect in rice, and investigate their regulating roles in chitin synthesis by injecting larvae with dsRNA. The results showed that *TRE1* and *TRE2* had compensatory function, and the expression of each increased when the other was silenced. The total rate of insects with phenotypic deformities ranged from 19.83 to 24.36% after dsTRE injection, whereas the mortality rate ranged from 14.16 to 31.78%. The mRNA levels of genes involved in the chitin metabolism pathway in RNA-Seq and DGEP, namely hexokinase (*HK*), glucose-6-phosphate isomerase (*G6PI*) and chitinase (*Cht*), decreased significantly at 72 h after single dsTREs injection, whereas two transcripts of chitin synthase (*CHS*) genes decreased at 72 h after dsTRE1-1 and dsTREs injection. These results demonstrated that *TRE* silencing could affect the regulation of chitin biosynthesis and degradation, causing moulting deformities. Therefore, expression inhibitors of *TRE*s might be effective tools for the control of planthoppers in rice.

Trehalose is a non-reducing disaccharide, formed by two glucose molecules linked by a 1α–1α bond, and is widely found in bacteria, fungi, plants, and invertebrates^1^. It is also called ‘blood sugar’, since it is an important energy substance of insect haemolymph and appears in all the developmental stages, including larva, pupae, and adults^2^,^3^,^4^,^5^. Trehalase (TRE; EC 3.2.1.28) is an anomer-inverting α-trehalose-1-D-glucosidase that hydrolyses a trehalose molecule into two glucose molecules. Two forms of TRE, soluble TRE (TRE1) and membrane-bound TRE (TRE2), have been identified and cloned in many insect species^6–14^.

TRE regulates homeostasis and development and is involved in insect energy metabolism, flight metabolism, growth, stress recovery, and chitin synthesis during moulting^10^,^11^,^15^,^16^,^17^,^18^,^19^,^20^. TRE1 is located within the cell and hydrolyses intracellular trehalose, whereas TRE2 is a trans-membrane enzyme with an active site on the outer side of the cell membrane that mainly hydrolyses extracellular trehalose^21^,^22^. Previous studies reported an increase in the mRNA levels of *TRE1*, but no effect on those of *TRE2* after the injection of hormone 20-hydroxyecdysone (20E) in *Bombyx mori*, suggesting that insect moulting is more closely related to *TRE1* than *TRE2*^21^. Other studies showed that the mRNA levels of *TRE1* increase before insect moulting and after the injection of 20E^21^,^23^,^24^. In *Apolygus lucorum*, ecdsone receptor (EcR) isoform-B mediates the expression of *TRE1* expression and regulates growth and development^25^.

*TRE1* and *TRE2* are known to regulate wing development, light metabolism, and chitin biosynthesis^13^,^20^. The chitin biosynthesis pathway involves eight enzymes, namely TRE, hexokinase (HK), glucose-6-phosphate isomerase (G6PI), fructose-6-phosphate transaminase (GFAT), glucosamine-phosphate N-acetyltransferase (GNPNA), phosphoacetylglucosamine mutase (PGM), UDP-N-acetylglucosamine pyrophosphorylase (UAP), and chitin synthase (CHS), and is crucially important for insect life^26^,^27^,^28^,^29^,^30^. CHS1 and CHS 2 are responsible for the synthesis of epidermal cuticle and midgut peritrophic matrix in various insects^30–35^. *TRE* regulates the chitin biosynthesis pathway by decreasing the expression of some of the involved genes, whereas chitinase (*Cht*) or chitinase-like genes are responsible for the degradation of chitin and thus, the completion of moulting^13^,^36^.

Rice (*Oryza sativa* L.), one of the world’s most important food crops worldwide, is attacked by 800 different insect species in the field and during storage^37^. The hemimetabolous brown planthopper *Nilaparvata lugens* Stål (Hemiptera: Delphacidae) is one of the most destructive insect pests of rice that causes significant yield losses^36^. RNA interference (RNAi), which is a robust and powerful experimental tool, has been widely used to study gene functions through gene silencing^34^ in various piercing-sucking insects, including rice planthoppers^36^,^38^,^39^,^40^,^41^. It has been reported that *TRE* regulates the expression of *CHS* in the cuticle and midgut of planthoppers, and the inhibition of chitin synthesis by suppressing or knocking down *TRE* leads to abnormal moulting and mortality. Therefore, we hypothesised that *TRE* controls the process of moulting by regulating the genes involved in the chitin biosynthesis pathway and accordingly, we aimed to study the functions and relationships of three TRE genes (*TRE 1-1*, *TRE 1-2*, and *TRE-2*) which were found from the genome of *N. lugens*^42^, as well as their regulating roles in chitin synthesis using RNAi and RNA-Sequencing (RNA-Seq).

## Results

### Relative expression of *TRE*s, activity of TREs, and trehalose content

**Figure 1:**
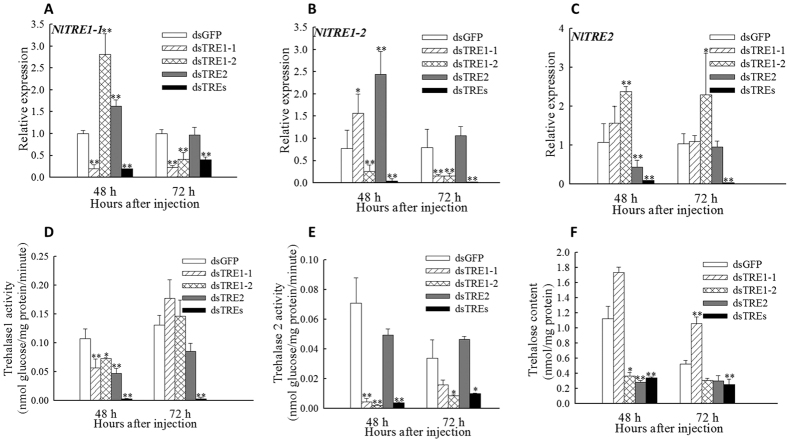
Relative expression of three trehalase (TRE) genes, activity of TRE1 and TRE2, and content of trehalose after double stranded RNA (dsRNA) injection of *Nilaparvata lugens* fifth instar larvae. (**A–C**) Changes in *TRE1-1*, *TRE1-2*, and *TRE2* expression at 48 h and 72 h after dsTRE1-1, dsTRE1-1, dsTRE2, dsTREs, and dsGFP injection. (**D**,**E**) Changes in TRE1 and TRE2 activity at 48 h and 72 h after dsTRE1-1, dsTRE1-1, dsTRE2, dsTREs, and dsGFP injection. (**F**) Changes in trehalose content at 48 h and 72 h after dsTRE1-1, dsTRE1-1, dsTRE2, dsTREs, and dsGFP injection. Bars represent means. Error bars indicate one standard error of the mean. ‘*’ Indicates significant differences at *p* < 0.05, and ‘**’ indicates significant differences at *p* < 0.01. Green fluorescence protein (GFP) was used as control.

The relative expression of *TRE1-1* and *TRE1-2* decreased significantly (*p* < 0.01) at 48 h and 72 h after dsTRE1-1 and dsTRE1-2 RNA injection (Fig. 1A,B) and that of *TRE2* at 48 h after dsTRE2 injection (Fig. 1A–C). *TRE1* and *TRE2* had compensatory function, because the expression of each increased when the other gene was silenced (Fig. 1). Besides, *TRE1-1*, *TRE1-2* and *TRE2* were all showed the super-low level which also indicated the crucial interaction of the three TREs (Fig. 1A–C). The activity of TRE1 decreased significantly (*p* < 0.05) at 48 h after dsTRE injection, but increased at 72 h after dsTRE1-1 and dsTRE1-2 injection (Fig. 1D). The activity of TRE2 decreased significantly (*p* < 0.01) at 48 h after dsTRE1-1 and dsTRE1-2 injection and at 72 h after dsTRE1-2 injection (Fig. 1E). Compared with the control (green fluorescence protein, GFP), the content of trehalose decreased significantly (*p* < 0.05) at 48 h after dsTRE1-2 and dsTRE2 injection and increased at 72 h after dsTRE1-1 injection (Fig. 1F). When knockdown all TREs, not only the activity of membrane bound as well as soluble trehalase but also the trehalose content displayed remarkable difference compared to control at 48 h and 72 h after injection (Fig. 1F).

### Characteristics of cDNA libraries, read annotation, and mapping

The characteristics of dsTRE1-1, dsTRE1-2, dsTRE2, and dsGFP cDNA libraries are presented in Table S1. The sequencing depth of dsTRE1-1, dsTRE1-2, dsTRE2, and dsGFP cDNA libraries was 12,464,195, 12,077,797, 11,803,689, and 11,953,979 reads, respectively, whereas the number of clean reads was 12,392,297, 12,002,051, 11,717,857, and 11883210, respectively. Saturation analysis indicated that the identified genes increased with the number of total reads.

All clean reads with 2-bp mismatch were mapped to the *N. lugens* reference genome and genes, and the results are presented in Table S2. A total of 9,288,949 (74.96%), 8,948,671 (74.56%), 8,829,104 (75.35%), and 9,079,399 (76.41%) clean reads in dsTRE1-1, dsTRE1-2, dsTRE2, and dsGFP cDNA libraries, respectively, was mapped to the reference genome, whereas a total of 4,107,840 (33.15%), 3,862,194 (32.18%), 3,599,258 (30.72%), and 3,618,777 (30.45%) clean reads, respectively, was mapped to the reference genes. Among these reads, 54.91%, 54.58%, 54.06%, and 54.46% in dsTRE1-1, dsTRE1-2, dsTRE2, and dsGFP cDNA libraries, respectively, distinctly matched, whereas 25.04%, 25.44%, 25.44%, and 23.59%, respectively, remained unmapped.

### Gene Ontology (GO) analysis and Digital Gene Expression Profiling (DGEP) of differentially expressed genes (DEGs)

**Figure 2:**
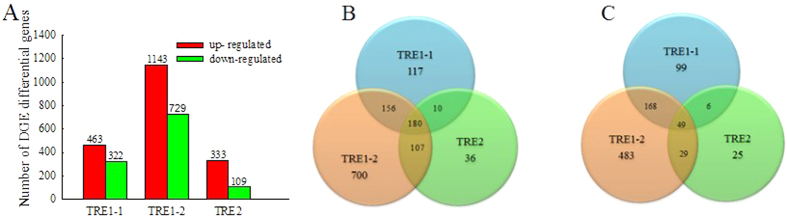
Differentially expressed genes after double stranded RNA (dsRNA) injection of *Nilaparvata lugens* fifth instar larvae for silencing three trehalase genes. (**A**) Differentially expressed genes after dsTRE1-2, dsTRE1-2, and dsTRE2 injection. (**B**) The Venn diagram of differentially expressed up-regulated genes after dsTRE1-2, dsTRE1-2, and dsTRE2 injection. (**C**) The Venn diagram of differentially expressed down-regulated genes after dsTRE1-2, dsTRE1-2, and dsTRE2 injection.

**Figure 3:**
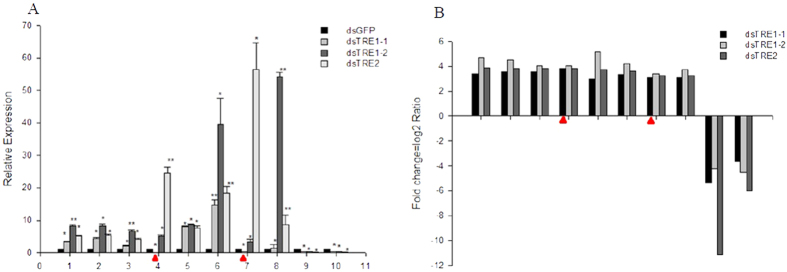
Expression of differentially expressed genes detected by quantitative real-time PCR (qRT-PCR) and Digital Gene Expression Profiling (DGEP) after double stranded RNA (dsRNA) injection of *Nilaparvata lugens* fifth instar larvae for silencing three trehalase genes. (**A**) Relative expression of candidate genes after dsTRE1-2, dsTRE1-2, dsTRE2, and dsGFP injection detected by qRT-PCR. (**B**) Fold change of candidate genes after dsTRE1-2, dsTRE1-2, and dsTRE2 injection detected by DGEP. 1-10 indicates the candidate genes, namely NLU011657.1, NLU028545.1, NLU009213.1, NLU020508.1, NLU027773.1, NLU002851.1, NLU005014.1, NLU012529.1, NLU015110.1, and NLU009094.1. Bars represent means. Error bars indicate one standard error of the mean. ‘*’ Indicates significant differences at *p* < 0.05, and ‘**’ Indicates significant differences at *p* < 0.01. Genes with different expression regulation are indicated by diamonds. Green fluorescence protein (GFP) was used as control.

The threshold with a false discovery rate (FDR) of ≤0.001 and a fold change ≥2 (absolute log2 Ratio ≥1) was used to identify DEGs (Fig. 2). Compared with dsGFP cDNA library, a total of 785, 1872, and 442 significant DEGs were identified in dsTRE1-1, dsTRE1-2, and dsTRE2 cDNA libraries, respectively, and of those 463, 1143, and 333 were up-regulated, whereas 322, 729, and 109 were down-regulated, respectively (Fig. 2A). The number of common DEGs that were up-regulated in the three dsTRE cDNA libraries was 180, whereas the number of those down-regulated was 49 (Fig. 2B,C). The number of unique DEGs that were up-regulated in dsTRE1-1, dsTRE1-2, and dsTRE2 cDNA libraries was 117, 700, and 36, respectively, whereas the number of those down-regulated was 99, 483, and 25, respectively (Fig. 2B,C). To confirm DGEP, semi-quantitative real-time PCR (semi qRT-PCR) was performed for 10 randomly selected genes of the 229 common DEGs. The results showed that eight genes had a concordant direction of change for both DGEP and qRT-PCR in the three dsTRE cDNA libraries, except for NLU020508.1 and NLU005014.1 in dsTRE1-1 (Fig. 3A,B).

**Figure 4:**
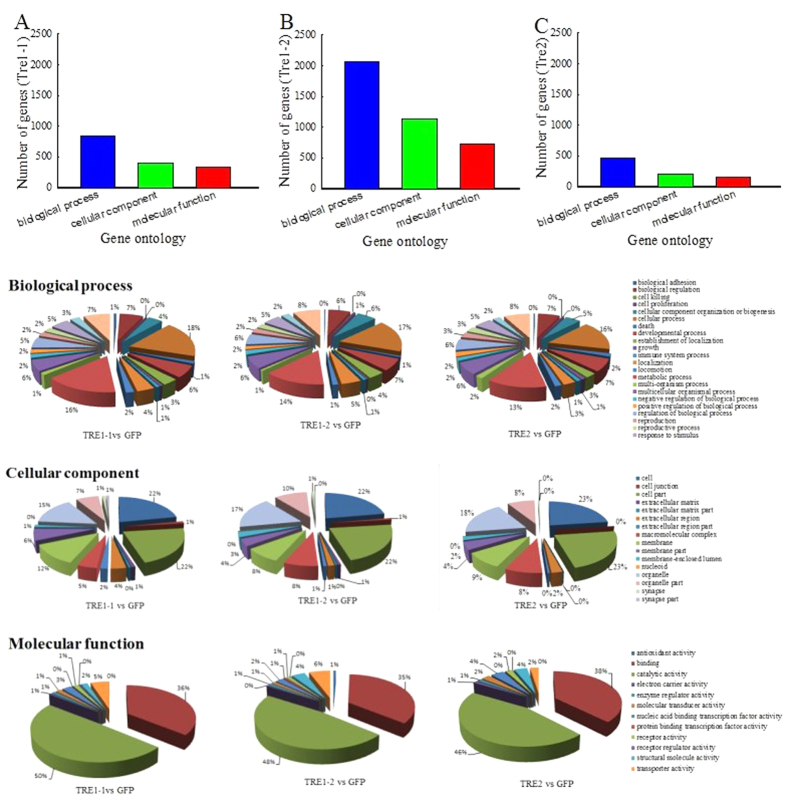
Digital Gene Expression Profiling (DGEP) and transcriptome comparative analysis of differentially expressed genes after double stranded RNA (dsRNA) injection of *Nilaparvata lugens* fifth instar larvae for silencing three trehalase genes. Median-normalized expression levels sorted by fold-change. Up-regulated false discovery rate (FDR) ≤ 0.001, log 2 Ratio ≥1. Down-regulated FDR ≤ 0.001, log 2 Ratio ≤ −1). Gene Ontology (GO) analysis was performed using WEB Gene Ontology Annotation Plot (WEGO).

GO analysis showed that the number of DEGs in dsTRE1-2 cDNA library was higher than that in dsTRE1-1 and dsTRE2 (Fig. 4B). In dsTRE1-2 cDNA library, 2,059 DEGs were assigned to ‘Biological Process’, including cellular process (17%) and metabolic process (14%); 726 DEGs were assigned to ‘Cellular Component’, including cell part (22%), cell (22%), organelle (17%), membrane (8%), and macromolecular complex (8%); and 1,142 DEGs were assigned to ‘Molecular Function’, including catalytic activity (48%), binding (35%), molecular transducer activity (6%), and structural molecule activity (4%) (Fig. 4). In dsTRE1-1 and dsTRE2 cDNA libraries, 845 and 462 DEGs were assigned to ‘Biological Process’, 335 and 162 DEGs to ‘Cellular Component’, and 406 and 201 DEGs to ‘Molecular Function’, respectively (Fig. 4).

### Pathway enrichment analysis using the Kyoto Encyclopaedia of Genes and Genomes (KEGG) database

**Figure 5:**
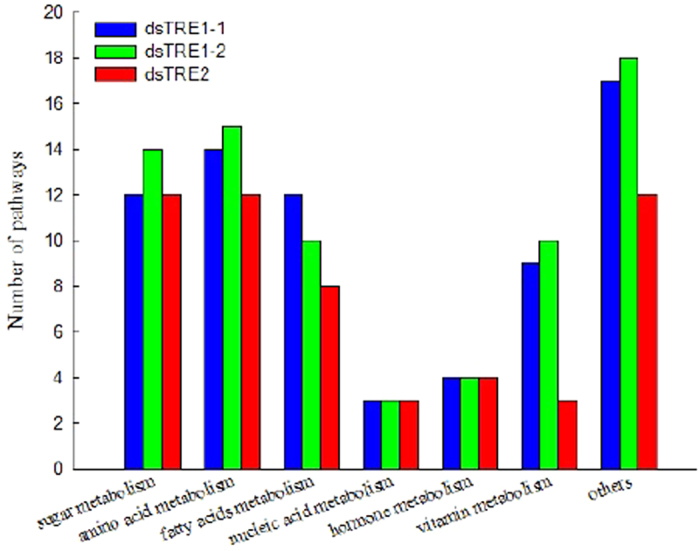
Pathway enrichment analysis using the Kyoto Encyclopaedia of Genes and Genomes (KEGG) database after double stranded RNA (dsRNA) injection of *Nilaparvata lugens* fifth instar larvae for silencing three trehalase genes. A Q value ≤ 0.05 was the criterion to standardise the significantly enriched pathways in differentially expressed genes.

Pathway enrichment analysis was used to identify significantly enriched metabolic pathways or signal transduction pathways in the three dsTRE cDNA libraries using KEGG database. The top 10 significantly enriched metabolic pathways are presented in Table S3. The number of enriched pathways related to sugar metabolism, amino acid metabolism, and vitamin metabolism, but not to fatty acid metabolism, was higher in dsTRE1-1 cDNA library than that in dsTRE1-2 and dsTRE2 (Fig. 5). The common enriched pathways in dsTRE1-1, dsTRE1-2, and dsTRE2 libraries were related to metabolic pathways, amoebiasis, influenza A, and focal adhesion processes. The number of DEGs in dsTRE1-2 cDNA library was higher than that in dsTRE1-1 and dsTRE2.

### The hierarchical clustering of expression patterns and key genes screen of differently expressed genes after three TRE RNAi

**Figure 6:**
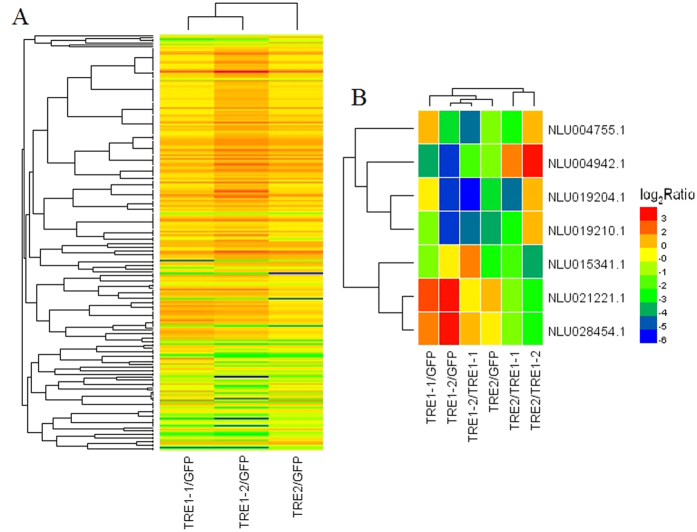
Hierarchical clustering of expression patterns of differently expressed genes after double stranded RNA (dsRNA) injection of *Nilaparvata lugens* fifth instar larvae for silencing three trehalase genes. (**A**) Heat map of expression patterns at 48 h after dsTRE1-1, dsTRE1-1, and dsTRE2 injection. (**B**) Seven common genes with different function identified at 48 h after dsTRE1-1, dsTRE1-1, and dsTRE2 injection. Clusters were constructed using Spearman rank-correlation coefficients on the trimmed mean of M (TMM)-normalised counts on each of the dsTRE1-1, dsTRE1-1, and dsTRE2 cDNA libraries. Red and green colours represent down-regulation and up-regulation, respectively.

The 229 DEGs (180 up-regulated and 49 down-regulated) showed a high degree of coincidence in dsTRE cDNA libraries (Figs 2 and 6). When this coincidence was extended to the type of regulation, it was revealed that most DEGs were regulated in the same way and with the similar strength in more than one dsTRE cDNA libraries. The results also showed the preferential involvement of different combinations of silencing proteins, based on the type of regulation and the growth phase of insect (Fig. 6, Table 1). About 22 genes were screened and identified to be related to energy metabolism, juvenile-hormone, vitellogenin, and heat shock proteins (Table 1). Of these, 15 genes were up-regulated and four genes were down-regulated in all three dsTRE cDNA libraries. A total of seven genes, including two HSP, two vitellogenin, one adenylate cyclase, one hypothetical secreted histidine rich peptide precursor, and one E3 ubiquitin-protein ligase SIAH1, showed different functions in the three dsTRE cDNA libraries (Fig. 6B).

### Morphology, mortality, and expression of genes involved in the chitin biosynthesis pathway after dsTRE and dsTREs injection

**Figure 7:**
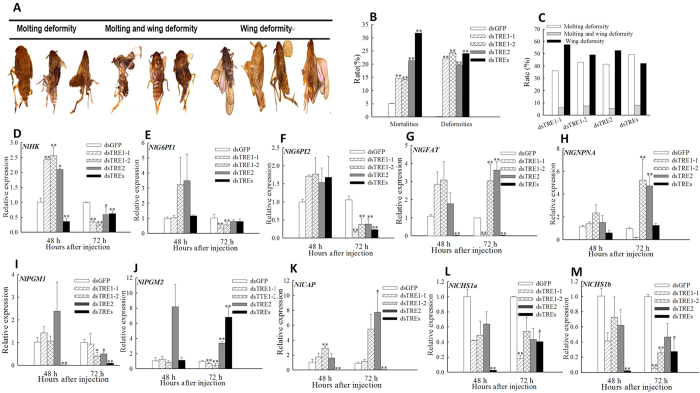
Phenotypic deformities and expression of genes involved in the chitin biosynthesis pathway after double stranded RNA (dsRNA) injection of *Nilaparvata lugens* fifth instar larvae for silencing three trehalase genes. (**A**) Larval phenotypes after dsTRE1-1, dsTRE1-1, dsTRE2, and dsTREs injection. (**B**) Rate of insects with phenotypic deformities and mortality rates after dsTRE1-1, dsTRE1-1, dsTRE2, dsTREs, and dsGFP injection. (**C**) Rate of insects with three different types of phenotypic deformities after dsTRE1-1, dsTRE1-1, dsTRE2, dsTREs, and dsGFP injection. (**D–M**) Relative expression of genes involved in the chitin biosynthesis pathway at 48 h and 72 h after dsTRE1-1, dsTRE1-1, dsTRE2, dsTREs, and dsGFP injection detected by quantitative real-time PCR. Bars represent means. Error bars indicate one standard error of the mean. ‘*’ Indicates significant differences at *p* < 0.05, and ‘**’ Indicates significant differences at *p* < 0.01. Genes with different expression regulation are indicated by diamonds. Green fluorescence protein (GFP) was used as control.

Insect phenotypes and mortality rates were estimated at 72 h after dsTRE injection, and phenotypic deformities were classified into three categories: moulting deformities, wing deformities, and moulting and wing deformities (Fig. 7A). The total rate of insects with phenotypic deformities and the mortality rate were 22.04% and 14.76% after dsTRE1-1 injection; 24.36% and 14.16% after dsTRE1-2 injection; and 19.83% and 21.43% after dsTRE2 injection, respectively (Fig. 7B). Interestingly, when all TREs were all silenced the rate of mortality and deformity were 31.78% and 24.11% which were higher (Fig. 7B). The total rates of moulting deformities, wing deformities, and moulting and wing deformities were 36.32%, 6.15%, and 57.53% after dsTRE1-1 injection; 42.98%, 49.28%, and 7.74% after dsTRE1-2 injection; and 41.62%, 5.56%, and 52.83% after dsTRE2 injection, respectively (Fig. 7C). The total rates of moulting deformities, wing deformities, and moulting and wing deformities were 49.36%, 8.33%, 42.31% when the three trehalase were knockdown together and the tendency is similar to single gene silenced (Fig. 7C).

The mRNA levels of genes involved in the chitin biosynthesis pathway after dsTRE injection were detected by qRT-PCR. The mRNA levels of *HK* increased significantly at 48 h, whereas those of *HK* and *G6PI2* decreased significantly (*p* < 0.05) at 72 h after dsTRE injection (Fig. 7D,F). Additionally, the mRNA levels of *GFAT*, *GNPNA*, and *UAP* increased significantly (*p* < 0.05), whereas *PGM1* decreased at 72 h after dsTRE1-2 and dsTRE2 injection (Fig. 7G,H,I,K). The fold change of *HK*, *GFAT*, *PGM1* and *UAP* was declined significantly (*p* < 0.01) at 48 h after all TREs were disturbed but there was no significance or significant difference when disturbed respectively (Fig. 7D,G,I,K). The relative expression levels of *HK*, *G6PI2* and *PGM1* at 72 h post injection were all decreased (*p* < 0.01) and the change is same when TREs were silenced separately or together (Fig. 7D,F). *NlCHS1* have two transcripts *CHS1a* and *CHS1b* (Supplemental Fig. S1)^38^, and the mRNA levels of *CHS1a* and *CHS1b* decreased significantly (*p* < 0.05) at 48 h and 72 h after dsTREs injection (Fig. 7L,M). As well as *CHS1a* and *CHS1b* decreased significantly (*p* < 0.05) at 72 h after dsTRE1-1 injection (Fig. 7L,M).

### Expression of *Cht* and chitinase-like genes after dsTRE and dsTREs injection

**Figure 8:**
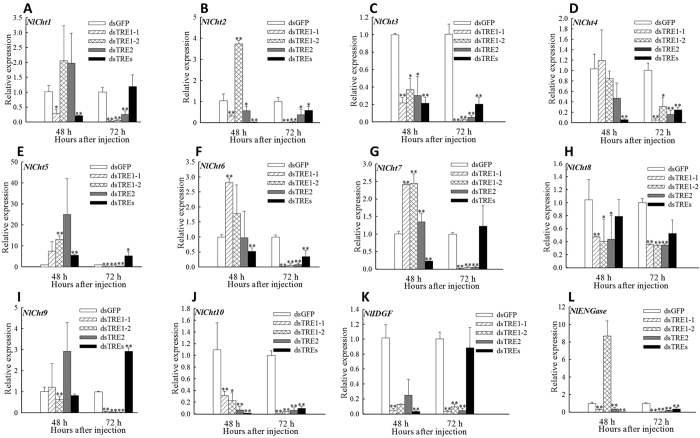
Relative expression of chitinase and chitinase-like genes after double stranded RNA (dsRNA) injection of *Nilaparvata lugens* fifth instar larvae for silencing three trehalase genes. (**A–L**) Expression of ten chitinase genes, one imaginal disc growth factor (*IDGF*), and one endo-β-N-acetylgucosaminidase (*ENGase*) at 48 h and 72 h after dsTRE1-1, dsTRE1-1, dsTRE2, dsTREs, and dsGFP injection detected by quantitative real-time PCR. Bars represent means. Error bars indicate one standard error of the mean. ‘*’ Indicates significant differences at *p* < 0.05, and ‘**’ Indicates significant differences at *p* < 0.01. Genes with different expression regulation are indicated by diamonds. Green fluorescence protein (GFP) was used as control.

The mRNA levels of *Cht* and chitinase-like genes, including 10 *Cht*, one imaginal disc growth factor (*IDGF*), and one endo-β-N-acetylgucosaminidase (*ENGase*), after dsTRE injection were detected by qRT-PCR (Fig. 8). The mRNA levels of *Cht3* and *Cht10* decreased significantly (*p* < 0.05) at 48 h after dsTRE injection and those of almost all 12 *Cht* or chitinase-like genes at 72 h. The mRNA levels of *Cht2*, *Cht5*, *Cht7*, *IDGF*, *ENGase* increased significantly (*p* < 0.01) at 48 h after dsTRE1-2 injection and those of *Cht7* (*p* < 0.01) after dsTRE2 injection. The expression of *Cht1*, *Cht2, Cht3*, *Cht4*, *Cht6*, *Cht7*, *Cht10*, *IDGF* and *ENGase* all showed a significant (*p* < 0.01) decrease at 48 h post injection of dsTREs. The relative fold change of *Cht2*, *Cht3*, *Cht4*, *Cht6*, *Cht10* and *ENGase* was also dramatically (*p* < 0.01) down-regulated.

## Discussion

Gene silencing by single-stranded RNA (siRNA) or dsRNA injection has been widely used to investigate gene function in insects^38^,^39^,^43^,^44^,^45^. In the present study, we investigated the relative expression of *TRE1* and *TRE2* after dsTRE and dsTREs injection and found that they have selective, discriminate, and compensatory functions; however, *TRE1-1* and *TRE1-2* probably have similar functions^3^,^8^. The relative expression of *TRE1-1* and *TRE1-2* decreased at 48 h and 72 h after dsTRE1-1 and dsTRE1-2 RNA injection and of *TRE2* at 48 h after dsTRE2 injection (Fig. 1A–C). These results were in agreement with those obtained in *Spodoptera exigua* and showed that *TRE1* increased at 12 h and *TRE2* at 24 h after dsTRE2 or dsTRE1 injection, respectively^13^. Our results also showed that the activity of TRE1 decreased at 48 h and of TRE2 at 48 h and 72 h after dsTRE1 injection, but the activity of TRE1 did not change after dsTRE1 injection and of TRE2 increased at 72 h after dsTRE2 injection (Fig. 1D,E). These results were in disagreement with those obtained in *S. exigua* and showed that the activity of TRE1 and TRE2 decreases after dsTRE1 or dsTRE2 injection, respectively^13^. It is possible that *N. lugens* other TREs have supplementary function when one TRE expression knockdown, and all of three TRE genes’ expression and trehalase activity decreased significantly at 48 h and 72 h after dsTREs injection (Fig. 1A–E).

Our results also showed that the concentration of TRE decreased after dsTRE1-2 and dsTRE2 injection, but increased after dsTRE1-1 injection (Fig. 1F), indicating that although the silencing of *TRE1-1* did not decrease the concentration of TRE, it could affect gene expression and lead to moulting deformities^13^ (Fig. 7A). Interestingly, we performed the knockdown of the three trehalases together and found some interesting points. Firstly, we find that the TRE1-1, TRE1-2 and TRE2 were all the super-low level. Secondly, the activity of membrane bound and soluble trehalase showed significant decrese. Thirdly, the content of trehalose also displayed dramatically down-regulated compared to control at 48 h and 72 h after injection. The above three points illustrate the important regulatory roles between TRE1 and TRE2 (Fig. 1A–F) and it showed that *N. lugens* have three TRE genes. Overall, these results revealed that expression inhibitors of *TRE*s might be an effective tool for the control of planthoppers in rice^20^,^46^.

RNA-Seq is considered a powerful tool for simultaneous transcriptome characterization, whereas DGEP helps to better understand the eco-physiological adaptations of insects^47^,^48^,^49^. Additionally, comparative transcriptome analysis is an effective way to identify DEGs and their functions under different conditions or treatments^50^,^51^. In this study, we combined RNA-Seq, DGEP, and comparative transcriptome analysis and identified a total of 229 DEGs that either up-regulated (180) or down-regulated (49) in all three dsTRE cDNA libraries (Fig. 2B,C). GO analysis showed that the number of DEGs in dsTRE1-2 cDNA library was higher than that in dsTRE1-1 and dsTRE2 (Figs 2A and 4). Overall, RNAi combined with RNA-Seq or DGEP is a functional approach to study gene function, screen downstream pathways, and identify DEGs in various biological systems^52^,^53^.

RNA-Seq and DGEP can also reveal different gene functions and the corresponding regulating pathways^51^,^54^,^55^. In this study, the number of enriched pathways related to sugar metabolism, amino acid metabolism, and vitamin metabolism was higher in dsTRE1-1 cDNA library than that in dsTRE1-2 and dsTRE2 (Fig. 5). The results also showed that *TRE1-2* was probably the main gene involved in TRE metabolism. The comparison of identified DEGs in different dsTRE cDNA libraries revealed a high degree of coincidence, and the 229 common DEGs were analysed by hierarchical clustering of expression patterns (Fig. 6A). The results showed seven common DEGs with different functions in each dsTRE cDNA library (Fig. 6B).

One of the main requirements for developing an RNAi-mediated pest control strategy is the identification of specific target genes that have a significant impact on insect development or viability^55^,^56^,^57^,^58^,^59^,^60^. In addition, the way of induce RNAi expressing dsRNA in the host plant or transgene-mediated RNAi have developed to target the expression of insect genes and used for pest control strategy^61^,^62^,^63^,^64^,^65^. The injection of dsTRE1 and dsTRE2 resulted in mortality rates over 50% in *S. exigua*^13^, whereas the dsRNA feeding method resulted in mortality rates of 38.89% in *S. exigua* and 27.72% in *Laodelphax stritellus*^14^. In our study, the mortality rates were 15%, 21% and 32% after dsTRE1, dsTRE2 or dsTREs injection, repectively (Fig. 7B), indicating the effect of *TRE* silencing on insect viability differs between species. However, moulting deformities, wing deformities, or failure of the old cuticle to break down were common in most species^42^. These phenotypes resulted from the silencing of genes involved in the chitin biosynthesis pathway or chitin degradation-related genes, including *CHS*^30^,^33^,^38^,^66^, *Cht*^36^, chitin deacetylase gene^34^,^40^, and β-N-acetylhexosaminidase gene^41^. Except for defaults in chitin metabolism, *TRE* silencing in *Drosophila* leads to loss of the lamina and reduction of the medulla^67^.

It is well known that the chitin biosynthesis pathway is crucially important for insect growth and development^31^,^32^,^33^,^68^, and also that *CHS1* and *CHS2* can be regulated by *TRE1* and *TRE2*, respectively^13^. The results showed that the mRNA levels of all 12 *Cht* or *Cht*-like genes, HK, two *G6PI*, and three *CHS1* decreased at 72 h after dsTRE injection especially when TREs were silenced at the same time (Figs 7 and 8). The rate of death and deformity increased to 31.78% and 24.11% when all TREs were disturbed and almost the balance of all 12 Cht or Cht-like genes was influenced at 48 h and 72 h post injection. It was also indicated that *TRE1-1* could regulate the expression of *CHS1*, results that were in agreement with those obtained in *S. exigua*^13^. The abnormal moulting in *N. lugens* indicated that *TRE* could regulate the expression of genes involved in chitin biosynthesis and degradation. However, the silencing of different genes leads to different rates of insects with phenotypic deformities and mortality rates. For example, the silencing of *CHS* in *S. exigua* leads to 50% abnormal phenotypes^69^ and 37.27% mortality^66^, the silencing of *UAP* in *Bactrocera dorsalis* leads to 65% abnormal phenotypes^70^, and the silencing of *Cht1*, *Cht5*, *Cht7*, *Cht9*, and *Cht10* in *N. lugen* leads to 50% mortality^36^. Mortality rates can reach up to 57% after the injection of 1μg of validamycin to *N. lugen* larvae (unpublished data), confirming that the application of *TRE* inhibitors can be a promising tool for pest control^20^.

## Materials and Methods

### Insects

*N. lugens* were collected from rice fields located at the China National Rice Research Institute, Hangzhou, China, in 2013 and kindly provided by Professor Qiang Fu. Larvae were reared on fresh rice (*O. sativa* L. var. TN-1) seedlings planted in cement tanks (60 cm × 30 cm × 100 cm) from April to October and in a greenhouse or growth chamber during the winter^60^. Caterpillars were kept at 25 ± 1 °C, 60–70% relative humidity, and a photoperiod of 16 h/8 h light/dark.

### Cloning of *TRE* cDNAs and sequencing analysis

The sequences of *N. lugens* trehalase homologs were obtained from the National Centre for Biotechnology Information (NCBI; *TRE1-1*, FJ790319 and *TRE2*, GQ397451) and early transcriptome and genomic sequencing results (*TRE1-2*)^42^. Total RNA was extracted from the whole body of 1–3-d-old fifth instar larvae using TRIzol (Invitrogen, Carlsbad, CA, USA), following the manufacturer’s instruction. RNA integrity was assayed by electrophoresis using 1.2% agarose gels. A special Nanodrop 2000 spectrophotometer (Thermo Fisher Scientific, Waltham, MA, USA) was used to detect the RNA concentration and purity. The cDNA synthesis was carried out according to the instructions of the PrimeScript™ RT reagent Kit with gDNA Eraser (Takara, Kyoto, Japan). Then, cDNAs were obtained by reverse transcription PCR using specific primers that designed were based on the obtained sequences (Table 2). The PCR products were purified with a special gel purification reagent (Omega, USA) and cloned into the PMD18-T vector (Takara, Dalian, China) for sequencing at Shanghai Sunny Biotechnology Co., Ltd. The sequencing results were aligned using the Blast program at the NCBI (http://blast.ncbi.nlm.nih.gov).

### dsRNA synthesis and injection

dsRNA was synthesised by PCR and *in vitro* transcription. PCR was performed using specific primers, containing terminal 5′ T7 promoter sites and a sequence specific to common regions of *TRE1-1*, *TRE1-2*, and *TRE-2*. The thermal cycling conditions were as follows: 40 cycles at 95 °C for 30 s, 58 °C for 30 s, and 72 °C for 45 s and a last extension at 72 °C for 10 min^9^. Purified *TRE* amplicons were *in-vitro* transcribed using T7 RiboMax Express RNAi System (Promega, Madison, USA)^71^. *GFP* amplicon was used to prepare control dsRNA. Sense and anti-sense strands were first produced in two separate transcriptive reactions and then combined and annealed at 70 °C for 10 min and in ice for 20 min. The dsRNAs were obtained by precipitation with 95% ethanol and 3 M sodium acetate (pH 5.2), washing with 70% ethanol, air drying, and resuspending. The integrity and quantity of dsRNA were analysed using Nanodrop 2000 (Thermo Fisher Scientific, Wilmington, DE, USA) and agarose gel electrophoresis. We verified the sequence by sequencing (Invitrogen Corporation, Shanghai, China) and maintained at −80 °C until use. The purified dsRNA were slowly injected using 3.5 Drummond needles and the NARISHIGE IM-31 (Nikon, JAPAN). Phenol red and dsRNA were mixed for clear observation.

The expression level of TREs is high at first day of 5^th^ instar larvae especially *TRE1-2* (Supplemental Fig. S2), and fifth larvae are suitable for dsRNA injection experiments. A total of 200 ng of dsTRE1-1, dsTRE1-2, dsTRE2 was injected into the abdomen side of *N. lugens* larvae using a microinjector. Also total of 200 ng of three dsTREs mixture was injected into the same larvae individual according the relative *N. lugens* genes’ function study^42^,^72^. Also *N. lugens* first day of 5^th^ instar larvae injected with dsGFP were used as control. The relative silencing efficiency was calculated by the mRNA levels after dsRNA injection in relation to the control.

### cDNA library construction and high-throughput sequencing

Total RNA was extracted from the whole body of *N. lugens* larvae or adults using TRIzol (Invitrogen), following the manufacturer’s instructions. RNA concentrations were determined using a spectrophotometer^73^ and sent to the Beijing Genomics Institute (BGI, Shenzhen, China) when TREs expression were inhibited by the way of RNAi.

*N. lugens* were collected at 48 h and 72 h after dsRNA injection to perform DGEP. Four cDNA libraries, namely dsTRE1-1, dsTRE1-2, dsTRE2, and dsGFP, were constructed for DGEP, and three RNA samples of equal amount from each library were pooled. The integrity of pooled samples was detected using the Agilent 2100 Bioanalyzer (Agilent, Folsom, CA, USA).

Poly(A)s, containing mRNAs, were collected from total RNA using oligo (dT) magnetic beads, and contaminants were washed out by a series of low-salt solution. The purified RNA samples were dissolved with Tris-base buffer, precipitated with ethanol, and resolubilised. The first and second strand cDNAs were synthesized using Oligo (dT), RNase H, and DNA polymerase I. The Oligo (dT)-bound cDNA was digested with the restriction enzyme NlaIII, which recognizes and cuts off the CATG sites. The fragments apart from the 3′ cDNA fragments connected to Oligo (dT) were washed away, and the Illumina adaptor 1 was ligated to the 5′ cohesive ends. Mmel, an endonuclease with separated recognition site and cleavage site, was used to generate reads with adaptor 1. The Illumina adaptor 2 was ligated to the 3′ ends of reads to form a cDNA library with different adaptors. After PCR amplification and purification, the fragments were tested for quality and quantity using the Agilent 2100 Bioanalyzer (Agilent) and the ABI StepOnePlus Real-time PCR system (Applied Biosystems, Carlsbad, CA, USA). The qualified cDNA library was sequenced on the Illumina HiSeqTM 2000 platform (Illumina, San Diego, CA, USA).

### Analysis of DEGs

Clean reads were obtained by filtering adaptor-containing reads, low quality reads, and reads with unknown nucleotides. The clean reads with at least 2-bp mismatch were mapped to the *N. lugens* reference reads of genome sequence^42^. The clean reads that mapped to multiple reference genes were filtered, whereas the remaining reads were designated as unambiguous clean reads.

The gene expression level was calculated and standardized using the reads per Kb per million reads (RPKM) method. The threshold with an FDR of ≤0.001 and a fold change ≥2 (absolute log2 Ratio ≥1) was used to identify DEGs in dsTRE cDNA libraries. DEGs were used for pathway enrichment analysis, GO enrichment analysis, and functional annotation clustering. GO annotation results were visualised, plotted, and compared using WEB Gene Ontology Annotation Plot (WEGO)^74^.

All DEGs were mapped in the GO data base, applying the hypergeometric test and identifying significantly enriched GO terms with *p* ≤ 0.05^54^. Pathway enrichment analysis was used to further identify significantly enriched metabolic pathways or signal transduction pathways using KEGG database. A Q value of ≤0.05 was the criterion to standardise the significantly enriched pathways in DEGs. Cluster analysis of gene expression was carried out using Cluster and Java Treeview^75^,^76^. Expression values were used for differential expression analysis between dsGFP and dsTRE cDNA libraries using two R packages of DESeq and edgeR (The R Project, Vienna, Austria)^77^. To avoid infinite values, a value of 1 was added to the normalized count value of each gene with zero value before log2 transformation. Hierarchical clustering was performed using hclust package in R with Manhattan distance^56^,^78^.

### Determination of TRE activity and trehalose content

The activity of TRE was determined as described previously with some modifications^10^,^11^. Thirty larvae were homogenized with phosphate buffer (pH 7.0). The homogenate was centrifuged at 1,000 *g* and 4 °C for 20 min, and 350 μl of the supernatant was collected and centrifuged at 20,800 *g* and 4 °C for 60 min. The supernatant was used for determining the activity and concentration of TRE1, whereas the sediment was suspended in phosphate buffer (pH 7.0) for determining the activity and concentration of TRE2. A total of 60 μl of the supernatant or suspension was mixed with 165 μl of phosphate buffer and 75 μl of 40 mM trehalose (Sigma-Aldrich, St. Louis, MO, USA), incubated at 37 °C for 1 h, and then centrifuged at 12,000 *g* and 4 °C for 10 min. The activities of TRE1 and TRE2 were determined in 10 μl of the supernatant using the glucose Assay Kit (Sigma-Aldrich, St. Louis, MO, USA). The content of trehalose was determined using the anthrone-sulfuric acid method^79^. The concentration of TRE1 and TRE2 was determined using the Pierce^TM^ BCA protein Assay Kit (Pierce Biotechnology, Rockford, IL, USA), following the manufacturer’s instructions.

### Sample collection, phenotype observations, and qRT-PCR

The injected nymphs were reared 25 ± 1 °C, 60–70% relative humidity, and a photoperiod of 16 h/8 h light/dark. The mortality rates and morphological phenotypes of the insects were observed and determined using a stereomicroscope (Leica S8AP0Z4, Germany) following the dsRNA treatments.

Thirty to fifty dsRNA-injected larvae from each treatment were collected at 12 h, 24 h, 36 h, 48 h, 60 h, and 72 h after injection to observe insect phenotypes. The relative expression of unique genes at the mRNA level was assessed using Bio-Rad CFX96TM Real-Time PCR Detection System (Bio-Rad Laboratories, Hercules, CA, USA) and SYBR Premix Ex Taq (Takara, Japan).

The primers used for realtime PCR in this experiment are listed in (Tables 3 and 4) which we confirmed the specificity by the production of standard and melting curves. PCR was performed in a final volume of 20 μl, including 1 μl of each primer, 10 μl of SYBR buffer, 7 μl of ultrapure water, and 1 μl cDNA. The thermal cycling conditions were as follows: denaturation at 95 °C for 3 min, and 35 cycles at 95 °C for 10 min and 60 °C for 30 s. The 18S RNA was quantified as an internal control. Each gene was analysed in triplicate, and the relative gene expression was calculated by the 2^−ΔΔCT^ method^80^.

### Statistical analysis

The mRNA expression levels of dsGFP injection were used as controls. The data are presented as means ± standard error (SE) of three to six replicates. Significant differences were identified by one-way analysis of variance (ANOVA) in conjunction with Duncan’s new multiple range test at *p* < 0.05 or 0.01^81^.

## Additional Information

**How to cite this article**: Zhao, L. *et al.* Functional characterization of three trehalase genes regulating the chitin metabolism pathway in rice brown planthopper using RNA interference. *Sci. Rep.* **6**, 27841; doi: 10.1038/srep27841 (2016).

## Supplementary Material

Supplementary Information

## Figures and Tables

**Table 1:** The transcriptome expressional level of common regulated genes after double stranded RNA (dsRNA) injection of *Nilaparvata lugens* fifth instar larvae for silencing three trehalase genes.

Gene ID	log2 Ratio (TRE1-1/GFP)	log2 Ratio (TRE1-2/GFP)	log2 Ratio (TRE2/GFP)	Gene Name	Similar species
NLU004755.1	1.38	−3.22	−1.34	adenylate cyclase	*Anopheles gambiae*
NLU004942.1	−3.73	−5.25	−1.08	hypothetical secreted histidine rich peptide precursor	*Dipetalogaster maximus*
NLU015341.1	−1.01	1.25	−2.54	E3 ubiquitin-protein ligase SIAH1	*Pediculus humanus corporis*
NLU021221.1	2.91	4.13	1.65	heat shock 70kDa protein	
NLU028454.1	2.36	3.74	1.32	heat shock 70kDa protein	*Trialeurodes vaporariorum*
NLU019204.1	1.045	−5.45	−3.50	vitellogenin	*Nilaparvata lugens*
NLU019210.1	−1.01	−5.45	−3.77	vitellogenin	*Nilaparvata lugens*
NLU019208.1	−2.47	−3.38	−3.69	vitellogenin	*Nilaparvata lugens*
NLU006117.1	2.32	2.86	2.15	juvenile-hormone esterase	*Nilaparvata lugens*
NLU012421.1	1.65	2.35	1.90	juvenile-hormone esterase/carboxylesterase	*Laodelphax striatella*
NLU012422.1	2.38	2.84	2.39	carboxylesterase	*Laodelphax striatella*
NLU014493.1	2.01	2.96	2.64	carboxylesterase/juvenile-hormone esterase	*Laodelphax striatella*
NLU016028.1	1.65	2.56	2.01	juvenile hormone esterase	*Nilaparvata lugens*
NLU027866.1	2.64	2.43	1.68	acetyl-CoA carboxylase	*Acyrthosiphon pisum*
NLU027867.1	2.62	2.50	1.59	acetyl-CoA carboxylase	*Acyrthosiphon pisum*
NLU009806.1	3.01	2.18	1.50	4-hydroxyphenylpyruvate dioxygenase	*Glossina morsitans morsitans*
NLU009865.1	−2.51	−3.57	−1.46	glucuronosyltransferase	*Pediculus humanus corporis*
NLU011630.1	1.90	1.70	1.01	isocitrate dehydrogenase	*Danaus plexippus*
NLU018865.2	1.24	2.14	1.94	glycoprotein 3-alpha-L-fucosyltransferase	*Pediculus humanus corporis*
NLU022777.1	2.67	2.23	1.41	ATP citrate lyase	*Acyrthosiphon pisum*
NLU012529.1	3.12	3.73	3.24	serine/threonine-protein phosphatase 4 regulatory subunit 2	*Ixodes scapularis*
NLU004377.1	2.36	2.95	2.52	peritrophin-like protein 1	*Ctenocephalides felis*

**Table 2:** Primers used for double stranded RNA synthesis.

Primer Name	Forwad Primer (5′-3′)	Reverse Primer (5′-3′)	Length (bp)
NLTRE1-1	ATGTCCCAATGTGCCATTCACAG	TCACGTACCATTCAAGAATATGTC	1803
DSNL*TRE1-1*	GATGCAATCAAGGAGGTGTTATGGC	CGTATTCACCTCCACCTCCGT	451
DSNL*TRE1-1*-T7	T7-GATGCAATCAAGGAGGTGTTATGGC	T7-CGTATTCACCTCCACCTCCGT	
NLTRE1-2	ATGAAGGCAAAAAAACATGGTGAGGCC	CTATAAATGATGCATGAAACGTTTTTCC	1506
DSNL*TRE1-2*	AGATGAAGGCATGTGGTTCG	CATCGATTCGCCAACTGGTAAGC	321
DSNL*TRE1-2*-T7	T7-AGATGAAGGCATGTGGTTCG	T7- CATCGATTCGCCAACTGGTAAGC	
NLTRE2	ATGACGACTGTTAATCTCTTCACGGT	CTAGTCACATGGTTTTAGATCCTTC	1998
DSNL*TRE*2	CCAACTGCTATGACACCGACAAG	GGGTTCAGATCCTGCCGTCGCT	440
DSNL*TRE*2-T7	T7-CCAACTGCTATGACACCGACAAG	T7-GGGTTCAGATCCTGCCGTCGCT	
DSNL*GFP*	AAGGGCGAGGAGCTGTTCACCG	CAGCAGGACCATGTGATCGCGC	688
DSNL*GFP*-T7	T7-AAGGGCGAGGAGCTGTTCACCG	T7-CAGCAGGACCATGTGATCGCGC	

T7 sequence: GGATCCTAATACGACTCACTATAGG.

**Table 3:** Primers used for quantitative real-time PCR (qRT-PCR) of genes involved in the chitin metabolism pathway.

Gene	Genebank number	Forwad (5′-3′)	Reverse (5′-3′)
NLTRE1-1	FJ790319	GCCATTGTGGACAGGGTG	CGGTATGAACGAATAGAGCC
NLTRE1-2	KU556829	GATCGCACGGATGTTTA	AATGGCGTTCAAGTCAA
NLTRE2	GQ397451	TCACGGTTGTCCAAGTCT	TGTTTCGTTTCGGCTGT
NLHK	KU556830	GGTGCGAGAAGAAGTGAAG	GTGAAACCCATTGGTAGAGT
NLGPI1	KU556831	CCTGCCACCAGTCATAACCC	CTTGTGAGGAAGGATGCGTTT
NLGPI2	KU556832	ATCAGCCGTAGCCAAGCAC	AAGCCGATAGCAGACCACAAC
NLGFAT	KU556833	CCTCCCAGTTCATCTCGC	CCAAGTTCTTCAAACCCTTTAT
NLGNPNA	KU556834	TGAGCTGCTGAAGACACT	CCTGAATAACGGTGATGTA
NLPGM1	JF330414	AACGAGACGGTGGGAGAC	TCCTGGTAAGTGTTGAGCC
NLPGM2	KU556835	AGAGGAAGGTTGGGAGTG	CATAATTCGCGGAGATAAG
NLUAP	JF330415	ACGACAGATTAAAGCCGATAC	TACCTTGTCCACCAGCCA
NLCHS1	AEL88648	CCGCAAACGATTCCTACAGA	AGGTCCTTGACGCTCATTCC
NLCHS1a	JQ040014	TGTTCTTGCTACAACTCAATAAA	ACACCAATCCGATAGGCTC
NLCHS1b	JQ040013	GCTGTCTTTGCTTTCTTCAT	ACACCAATCCGATAGGCTC
NLCht1	AJO25036	AGGTGGTTAGGGACGAGGAG	TGCGCTTGACATAGTTGGACT
NLCht2	AJO25037	GCAGATTTCTGGACAGGGAA	TGACGCACAAGCGGGAAG
NLCht3	AJO25038	CTACACCTCTGGCTAAACTCGG	AACTTGTCCTTGCGGCTGAT
NLCht4	AJO25039	TTGAGGAGGTTCACGGGTCT	CCTTACTGGAAACGAGGTTGG
NLCht5	AJO25040	AAAGCGTTCGTGATGAAATAGC	GATCCTTTGCCTCAATCCAAT
NLCht6	AJO25041	GCTGGTAAGGAGATGCTATTCG	GTGGTTCTAAGGCTGGCTGTC
NLCht7	AJO25042	CTACTCTGCCATCCCATTCCT	GTCTGGGTTTCTTCACTTCCTG
NLCht8	AJO25043	GAACAAAGTGCAAACTCAGTCC	CACCTTCTGTGGCTTCTGG
NLCht9	AJO25044	GTGCGGTATTGGTTGAAGAGG	GGTATAACGTGATTCCGAGCC
NLCht10	AJO25045	CAAGCCAATACCCAACAAAC	ACAGCAAATCCATAGAGCACA
NLIDGF	AJO25056	AAAAGAACGAGGAGGAGGG	TTGCTTGAGGATGGGGTAC
NLENGase	AJO25057	TGTGGCAAGACTTCGTTA	ATGGGAGGGTTGGGATAG
NL-18S	–	CGCTACTACCGATTGAA	GGAAACCTTGTTACGACTT

**Table 4:** Primers used for the verification of Digital Gene Expression Profiling (DGEP) verification by PCR.

Primer Name	Gene	Forwad Primer (5′-3′)	Reverse Primer (5′-3′)
NLU011657.1	serine protease	GGCAGAGTTCGCTGTTTGG	CTTGCTGATTAGGATGGAGGC
NLU028545.1	–	TCACGGTATGCCAAGTCAAGTT	GCCTTCGTTTCAGTTCTCGTT
NLU009213.1	–	AGAAAGATAGCAGTGGCAAACC	CTCAACAGGGCAGCAATCAC
NLU020508.1	–	GTGAGGAGTGGGGATGTGCT	GACCAGGTGGAGGGAACTATGT
NLU027773.1	baculoviral IAP repeat-containing protein	AGTTTCAACTTTCCATCGTCCA	TCCAGCTTGTTTTCCCACAG
NLU002851.1	protein-tyrosine phosphatase	TGCTGGTGGAGACAGTGAGG	TGTTTGTGCGTTTCCAGTAGG
NLU005014.1	hypothetical protein SINV	CAAACTGGCTTGGTCGCA	GAAGACCCAATACCGATGTGC
NLU012529.1	serine/threonine-protein phosphatase 4 regulatory subunit 2	ACTGGCGGGATCAGGTGTA	GCTTCTTAACGTCGGGTTCTT
NLU015110.1	–	ATGGAAAGGACGAAACAGGAA	CTAGCAGTGAGACCAGGAGGAG
NLU009094.1	myosin-light-chain kinase	CCTGATGACTTTGCGGGATA	TGGATTGGGTTACTGGTTGACT

## References

[b1] Avonce N., Mendoza-Vargas A., Morett E. & Iturriaga G. Insights on the evolution of trehalose biosynthesis. BMC Evol Bio. 6, 109 (2006). 17178000 10.1186/1471-2148-6-109 PMC1769515

[b2] Wyatt G.-R. The biochemistry of sugars and polysaccharides in insects. Adv Insect Physiol 4, 287–360 (1967).

[b3] Tang B., Wei P., Chen J., Wang S.-G. & Zhang W.-Q. Progress in gene features and functions of insect trehalases. Acta Entomol Sin 55, 1315–1321 (2012).

[b4] Becker A., Schlder P., Steele J. E. & Wegener G. The regulation of trehalose metabolism in insects. Experientia 52, 433–439 (1996). 8706810 10.1007/BF01919312

[b5] Elbein A.-D., Pan Y.-T., Pastuszak I. & Carroll D. New insights on trehalose: a multifunctional molecule. Glycobiology 13, 17–27 (2003). 10.1093/glycob/cwg047 12626396

[b6] Takiguchi M., Niimi T., Su Z.-H. & Yaginuma T. Trehalase from male accessory gland of an insect, *Tenebrio molitor*. cDNA sequencing and developmental profile of the gene expression, Biochem J 288, 19–22 (1992). 1445264 10.1042/bj2880019 PMC1132073

[b7] Parkinson N.-M. *et al.* cDNAs encoding large venom proteins from the parasitoid wasp *Pimpla hypochondriaca* identified by random sequence analysis, Comp Biochem Physiol 134C, 513–520 (2003). 10.1016/s1532-0456(03)00041-3 12727301

[b8] Mitsumasu K., Azuma M., Niimi T., Yamashita O. & Yamashita T. Membrane-penetrating trehalase from silkworm *Bombyx mori*. Molecular cloning and localization in larval midgut. Insect Mol Biol 14, 501–508 (2005). 16164606 10.1111/j.1365-2583.2005.00581.x

[b9] Tang B. *et al.* Characterization and expression patterns of a membrane-bound trehalase from *Spodoptera exigua*. *BMC Mole* Biol 9, 51 (2008). 10.1186/1471-2199-9-51 PMC2424068 18492231

[b10] Tatun N., Singtripop T. & Sakurai S. Dual control of midgut trehalase activity by 20-hydroxyecdysone and an inhibitory factor in the bamboo borer *Omhisa fuscidentalis Hampson*. J Insect Physiol 54, 351–357 (2008). 18023454 10.1016/j.jinsphys.2007.10.006

[b11] Tatun N., Singtripop T., Tungjitwitayakul J. & Sakurai S. Regulation of soluble and membrane-bound trehalase activity and expression of the enzyme in the larval midgut of the bamboo borer *Omphisa fuscidentalis*. Insect Biochem Mol Biol 38, 788–795 (2008). 18625402 10.1016/j.ibmb.2008.05.003

[b12] Lee J.-H. *et al.* Molecular cloning of cDNA for trehalase from the European honeybee, *Apis mellifera L* and its heterologous expression in *Pichia pastoris*. Biosci Biotechnol Biochem 71, 2256–2265 (2007). 17827701 10.1271/bbb.70239

[b13] Chen J. *et al.* Different functions of the insect soluble and membrane-bound trehalase genes in chitin biosynthesis revealed by RNA Interference. PloS One 5, e10133 (2010). 20405036 10.1371/journal.pone.0010133 PMC2853572

[b14] Zhang Q., Lu D.-H., Pu J., Wu M. & Han Z.-J. Cloning and RNA interference effects of trehalase genes in Laodelphax striatellus (Homoptera: Delphacidae). Acta Entomol Sin 55, 911–920 (2012).

[b15] Clegg J.-S. & Evans D.-R. Blood trehalose and flight metabolism in the blowfly. Science 134, 54–55 (1961). 13694011 10.1126/science.134.3471.54

[b16] Friedman S. Trehalose regulation, one aspect of metabolic homeostasis. Annu Rev Entomol 23, 389–407 (1978).

[b17] Terra W.-R. & Ferreira C. Insect digestive enzymes: properties, compartmentalization and function. Comp Biochem Physiol 109B, 1–62 (1994).

[b18] Thompson S.-N. Trehalose—the insect ‘blood’ sugar. Adv Insect Physiol 31, 203–285 (2003).

[b19] Silva C.-P.-M., Terra R.-W. & Ferreira C. The role of carboxyl, guanidine and imidazole groups in catalysis by a midgut trehalase purified from an insect larva. Insect Biochem Mol Biol 34, 1089–1099 (2004). 15475303 10.1016/j.ibmb.2004.07.001

[b20] Shukla E., Thorat L.-J., Nath B.-B. & Gaikwad S.-M. Insect trehalase: physiological significance and potential applications. Glycobiology 25, 357–367 (2015). 25429048 10.1093/glycob/cwu125

[b21] Mitsumasu K., Azuma M., Niim T., Yamashita O. & Yaginuma T. Changes in the expression of soluble and integral-membrane trehalases in the midgut during metamorphosis in *Bombyx mori*. Zool Sci. 25, 693–698 (2008). 18828655 10.2108/zsj.25.693

[b22] Almeida F.-M. & Bonini B.-M. Heterologous expression in *Escherichia coli* of *Neurospora crassa* neutral trehalase as an active enzyme. Protei Expre Purif 65, 185–189 (2009). 10.1016/j.pep.2008.11.010 19073263

[b23] Kunieda T. *et al.* Carbohydrate metabolism genes and pathways in insects: insights from the honey bee genome. Insect Mol Biol 15, 563–576 (2006). 17069632 10.1111/j.1365-2583.2006.00677.x PMC1847477

[b24] Yao Q. *et al.* Identification of 20-Hydroxyecdysone Late-Response Genes in the Chitin Biosynthesis Pathway. PLOS One 5, e14058 (2010). 21124981 10.1371/journal.pone.0014058 PMC2987807

[b25] Tan Y.-A. *et al.* Ecdysone receptor isoform-B mediates soluble trehalase expression to regulate growth and development in the mirid bug, *Apolygus lucorum* (Meyer-Dür). Insect Mol Biol 24, 611–623 (2015). 26335337 10.1111/imb.12185

[b26] Candy D.-J. & Kilby B.-A. Studies on chitin synthesis in the desert locust. J Exp Biol 39, 129–140 (1962).

[b27] Kramer K.-J., Dziadik-Turner C. & Koga D. Chitin metabolism in insects. Comp. Insect Physiol Biochem Pharmacol 3, 75–115 (1985).

[b28] Kramer K.-J. & Muthukrishnan S. Chitin metabolism in insects. in Comprehensive Molecular Insect Science, Vol. 4 (eds Gilbert L. I. *et al.*) 111–144 (Elsevier, Oxford, 2005).

[b29] Zhang W.-Q. *et al.* Insect chitin biosynthesis and its regulation. Chin J Appl Entomol 48, 475–479 (2011).

[b30] Chen X.-F. *et al.* Disruption of *Spodoptera exigua* larval development by silencing chitin synthase gene A with RNA interference. Bull Entomol Res 98, 613e619 (2008). 18662430 10.1017/S0007485308005932

[b31] Arakane Y. *et al.* Characterization of two chitin synthase genes of the red flour beetle, *Tribolium castaneum*, and alternate exon usage in one of the genes during development. Insect Biochem Mol Biol 34, 291–304 (2004). 14871625 10.1016/j.ibmb.2003.11.004

[b32] Arakane Y. *et al.* The *Tribolium* chitin synthase genes TcCHS1 and TcCHS2 are specialized for synthesis of epidermal cuticle and midgut peritrophic matrix. Insect Mol Biol 14, 453e463 (2005). 16164601 10.1111/j.1365-2583.2005.00576.x

[b33] Arakane Y., Specht C.-A., Kramer K.-J., Muthukrishnan S. & Beeman R.-W. Chitin synthases are required for survival, fecundity and egg hatch in the red flour beetle, Tribolium castaneum. Insect Biochem Mol Biol 38, 959–962 (2008). 18718535 10.1016/j.ibmb.2008.07.006

[b34] Arakane Y. *et al.* Analysis of functions of the chitin deacetylase gene family in *Tribolium castaneum*. Insect Biochem Mol Biol 39, 355–365 (2009). 19268706 10.1016/j.ibmb.2009.02.002

[b35] Qu M. & Yang Q. A novel alternative splicing site of class A chitin synthase from the insect *Ostrinia furnacalise* Gene organization, expression pattern and physiological significance. Insect Biochem Mol Biol 41, 923–931 (2011). 21933709 10.1016/j.ibmb.2011.09.001

[b36] Xi Y. *et al.* Chitinase-like gene family in the brown planthopper, Nilaparvata lugens. Insect Mol Biol 24, 29–40 (2015). 25224926 10.1111/imb.12133

[b37] Barrion A.-T. & Litsinger J.-A. Taxonomy of rice insect pests and their arthropod parasites and predators. (ed. Heinrichs E. A.) Ch. 2, 13–362 (Wiley Eastern Ltd., India and IRRI, Manila, Philippines, 1994).

[b38] Wang Y. *et al.* Chitin synthase 1 gene and its two alternative splicing variants from two sap-sucking insects, *Nilaparvata lugens* and *Laodelphax striatellus* (Hemiptera: Delphacidae). Insect Biochem Mol Biol. 42, 637–646 (2012). 22634163 10.1016/j.ibmb.2012.04.009

[b39] Liu S. *et al.* RNA interference of NADPH-cytochrome P450 reductase of the rice brown planthopper, *Nilaparvata lugens*, increases susceptibility to insecticides. Pest Manag Sci. 71, 32–39 (2015). 24515640 10.1002/ps.3760

[b40] Xi Y., Pan P.-L., Ye Y.-X., Yu B. & Zhang C.-X. Chitin deacetylase family genes in the brown planthopper, *Nilaparvata lugens* (Hemiptera: Delphacidae). Insect Mol Biol 23, 695–705 (2014). 24989071 10.1111/imb.12113

[b41] Xi Y., Pan P.-L. & Zhang C.-X. The β-N-acetylhexosaminidase gene family in the brown planthopper, Nilaparvata lugens. Insect Mol Biol 24, 601–610 (2015). 26304035 10.1111/imb.12187

[b42] Xue J. *et al.* Genomes of the rice pest brown planthopper and its endosymbionts reveal complex complementary contributions for host adaptation. Genome Biol 15, 521 (2014). 25609551 10.1186/s13059-014-0521-0 PMC4269174

[b43] Belles X. Beyond *Drosophila*: RNAi *in vivo* and functional genomics in insects. Annu Rev Entomol 55, 111–128 (2010). 19961326 10.1146/annurev-ento-112408-085301

[b44] Lozano J. & Bellex X. Conserved repressive function of Kru¨ppel homolog 1 on insect metamorphosis in hemimetabolous and holometabolous species. Sci. Rep. 1, 163 (2011). 22355678 10.1038/srep00163 PMC3240953

[b45] Li K. *et al.* *Bombyx* E75 isoforms display stage- and tissue-specific responses to 20-hydroxyecdysone Sci. Rep. 5, 12114 (2015). 26166384 10.1038/srep12114 PMC4499807

[b46] Wegener G., Macho C., Schlöder P., Kamp G. & Ando O. Long-term effects of the trehalase inhibitor trehazolin on trehalase activity in locust flight muscle. J Exp Biol 213, 3852–3857 (2010). 21037064 10.1242/jeb.042028

[b47] Bonizzoni M. *et al.* RNA-seq analyses of blood-induced changes in gene expression in the mosquito vector species, Aedes aegypti. BMC Genomics 12, 82 (2011). 21276245 10.1186/1471-2164-12-82 PMC3042412

[b48] Pitts R.-J., Rinker D.-C., Jones P.-L., Rokas A. & Zwiebel L.-J. Transcriptome profiling of chemosensory appendages in the malaria vector *Anopheles gambiae* reveals tissue- and sex-specific signatures of odor coding. BMC Genomics 27, 271 (2011). 21619637 10.1186/1471-2164-12-271 PMC3126782

[b49] Mamidala P. *et al.* RNA-Seq and molecular docking reveal multi-level pesticide resistance in the bed bug. BMC Genomics 13, 6 (2012). 22226239 10.1186/1471-2164-13-6 PMC3273426

[b50] Ou J. *et al.* Transcriptomic analysis of developmental features of *Bombyx mori* wing disc during metamorphosis. BMC Genomics 15, 820 (2014). 25261999 10.1186/1471-2164-15-820 PMC4196006

[b51] Nascimento A.-R., Fresia P., Cônsoli F.-L. & Omoto C. Comparative transcriptome analysis of lufenuron-resistant and susceptible strains of *Spodoptera frugiperd*a (Lepidoptera: Noctuidae). BMC Genomics 16, 985 (2015). 26589731 10.1186/s12864-015-2183-z PMC4654862

[b52] Nizard P. *et al.* Integrative analysis of high-throughput RNAi screen data identifies the FER and CRKL tyrosine kinases as new regulators of the mitogenic ERK-dependent pathways in transformed cells. BMC Genomics 15, 1169 (2014). 25540073 10.1186/1471-2164-15-1169 PMC4367906

[b53] Yamaguchi U. *et al.* Functional genome screen for therapeutic targets of osteosarcoma. Cancer Sci. 100, 2268–2274 (2009). 19725836 10.1111/j.1349-7006.2009.01310.x PMC11158744

[b54] Ashburner M. *et al.* Gene ontology: tool for the unification of biology. The Gene Ontology Consortium. Nat Genet 25, 25–29 (2000). 10802651 10.1038/75556 PMC3037419

[b55] Camargo Rde.-A. *et al.* De novo transcriptome assembly and analysis to identify potential gene targets for RNAi-mediated control of thetomato leafminer (*Tuta absoluta*). BMC Genomics 16, 635 (2015). 26306628 10.1186/s12864-015-1841-5 PMC4550053

[b56] Nicolás F.-E. *et al.* The RNAi machinery controls distinct responses to environmental signals in the basal fungus *Mucor circinelloides*. BMC Genomics 16, 237 (2015). 25880254 10.1186/s12864-015-1443-2 PMC4417260

[b57] Guo Z. *et al.* The novel ABC transporter ABCH1 is a potential target for RNAi based insect pest control and resistance management. Sci. Rep. 5, 13728 (2015). 26333918 10.1038/srep13728 PMC4558546

[b58] Nie H. *et al.* Functional loss of *Bmsei* causes thermosensitive epilepsy in contractile mutant silkworm, Bombyx mori. Sci. Rep. 5, 12308 (2015). 26198671 10.1038/srep12308 PMC4510522

[b59] Ulrich J. *et al.* Large scale RNAi screen in *Tribolium* reveals novel target genes for pest control and the proteasome as primetarget. BMC Genomics 16, 674 (2015). 26334912 10.1186/s12864-015-1880-y PMC4559001

[b60] Zhang Y.-X. *et al.* RNAi knockdown of acetyl-CoA carboxylase gene eliminates jinggangmycin-enhanced reproduction and population growth in the brown planthopper, *Nilaparvata lugens*. Sci. Rep. 5, 15360 (2015). 26482193 10.1038/srep15360 PMC4611885

[b61] Baum J.-A. *et al.* Control of coleopteran insect pests through RNA interference. Nat Biotechnol 25, 1322–1326 (2007). 17982443 10.1038/nbt1359

[b62] Mao Y.-B. *et al.* Silencing a cotton bollworm P450 monooxygenase gene by plant-mediated RNAi impairs larval tolerance of gossypol. Nat Biotechnol 25, 1307–1313 (2007). 17982444 10.1038/nbt1352

[b63] Pitino M., Coleman A. D., Maffei M. E., Ridout C. J. & Hogenhout S. A. Silencing of aphid genes by dsRNA feeding from plants. PLoS One 6, e25709 (2011). 21998682 10.1371/journal.pone.0025709 PMC3187792

[b64] Zha W.-J. *et al.* Knockdown of midgut genes by dsRNA-transgenic plant-mediated RNA interference in the hemipteran insect *Nilaparvata lugens*. PLoS One 6, e20504 (2011). 21655219 10.1371/journal.pone.0020504 PMC3105074

[b65] Kumar B., Pandit S. S. & Baldwin I. T. Tobacco rattle virus vector: A rapid and transient means of silencing *Manduca sexta* genes by plant mediated RNA interference. PLoS One 7, e31347 (2012). 22312445 10.1371/journal.pone.0031347 PMC3270032

[b66] Tian H.-G. *et al.* Developmental regulation of a lepidopteran pest *Spodoptera exigua* by ingestion of bacteria expressing dsRNA of a non-midgut gene, PLoS One 4, e6225 (2009). 19593438 10.1371/journal.pone.0006225 PMC2704864

[b67] Chen X., Quan Y., Wang H. & Luo H. Trehalase regulates neuroepithelial stem cell maintenance and differentiation in the *Drosophila* optic lobe. PLoS One 9, e101433 (2014). 25003205 10.1371/journal.pone.0101433 PMC4086926

[b68] Zhu K.-Y., Merzendorfer H., Zhang W. Q., Zhang J. Z. & Muthukrishnan S. Biosynthesis, turnover, and functions of chitin in insects. Annu Rev Entomol 61, 177–196 (2016). 26982439 10.1146/annurev-ento-010715-023933

[b69] Chen X.-F. *et al.* The class A chitin synthase gene of *Spodoptera exigua*: Molecular cloning and expression patterns. Insect Biochem Mol Biol 37, 409–417 (2007). 17456436 10.1016/j.ibmb.2007.01.006

[b70] Yang W.-J. *et al.* Two chitin biosynthesis pathway genes in *Bactrocera dorsalis* (Diptera: Tephritidae): molecular characteristics, expression patterns, and roles in larval-pupal transition. J Econ Entomol 108, 2433–2442 (2015). 26453732 10.1093/jee/tov186

[b71] Wang J.-D., Wu M., Wang B.-J. & Han Z.-J. Comparison of the RNA interference effects triggered by dsRNA and siRNA in *Tribolium castameum*. Pest Manag Sci. 69, 781–786 (2013). 23526733 10.1002/ps.3432

[b72] Xu H.-J. *et al.* Two insulin receptors determine alternative wing morphs in planthoppers. Nature 519, 464–467 (2015). 25799997 10.1038/nature14286

[b73] Li J.-J. *et al.* RNA interference of the P450 CYP6CM1 gene has different efficacy in B and Q biotypes of *Bemisia tabaci*. Pest Manag Sci. 71, 1175–1181 (2015). 25200527 10.1002/ps.3903

[b74] Ye J. WEGO: a web tool for plotting Go annotations. Nucleic Acids Res 34, 293–297 (2006). 10.1093/nar/gkl031 PMC1538768 16845012

[b75] Eisen M.-B., Spellman P.-T., Brown P.-O. & Botstein D. Cluster analysis and display of genome-wide expression patterns. Proc. Natl. Acad. Sci. USA 95, 14863–14868 (1998). 9843981 10.1073/pnas.95.25.14863 PMC24541

[b76] Saldanha A.-J. Java Tree view—extensible visualization of microarray data. Bioinformatics 20, 324–3248 (2004). 10.1093/bioinformatics/bth349 15180930

[b77] Anders S. & Huber W. Differential expression analysis for sequence count data. Genome Biol 11, R106 (2010). 20979621 10.1186/gb-2010-11-10-r106 PMC3218662

[b78] Calla B., Hall B., Hou S. & Geib S.-M. A genomic perspective to assessing quality of mass-reared SIT flies used in Mediterranean fruit fly (*Ceratitis capitata*) eradication in California. BMC Genomics 15, 98 (2014). 24495485 10.1186/1471-2164-15-98 PMC3923235

[b79] Leyva A. *et al.* Rapid and sensitive anthrone-sulfuric acid assay in microplate format to quantify carbohydrate in biopharmaceutical products: method development and validation. Biologicals 36, 134–141 (2008). 18042399 10.1016/j.biologicals.2007.09.001

[b80] Livak K. J. & Schmittgen T. D. Analysis of relative gene expression data using real-time quantitative PCR and the 2-^−ΔΔCT^ method. Methods 25, 402–408 (2001). 11846609 10.1006/meth.2001.1262

[b81] Tang T., Zhao C.-Q., Feng X.-Y., Liu X.-Y. & Qiu L.-H. Knockdown of several components of cytochrome P450 enzyme systems by RNA interference enhances the susceptibility of *Helicoverpa armigera* to fenvalerate. Pest Manag Sci. 68, 1501–1511 (2012). 22689565 10.1002/ps.3336

