# Supplementary material for: Functional characterization of three trehalase genes regulating the chitin metabolism pathway in rice brown planthopper using RNA interference
Source: Sci Rep. 2016 Jun 22;6:27841. doi: 10.1038/srep27841 (PMC4916506; doi:10.1038/srep27841)
Supplement: Supplementary Information [file srep27841-s1.docx]

**Functional characterization of three trehalase genes regulating the chitin metabolism pathway in rice brown planthopper using RNA interference**

Lina Zhao, Mengmeng Yang, Qida Shen, Xiaojun Liu, Zuokun Shi, Shigui Wang, Bin Tang*

**Supplementary Figs**

**Fig. S1. The different sequences of** **NlCHS1a and 1b. We designed the** **NlCHS1a primer from 3931 position (TGTTCTTGCTACAACTCAATAAA,bottom)and NlCHS1b primer from 3885 position (GCTGTCTTTGCTTTCTTCAT,top)**


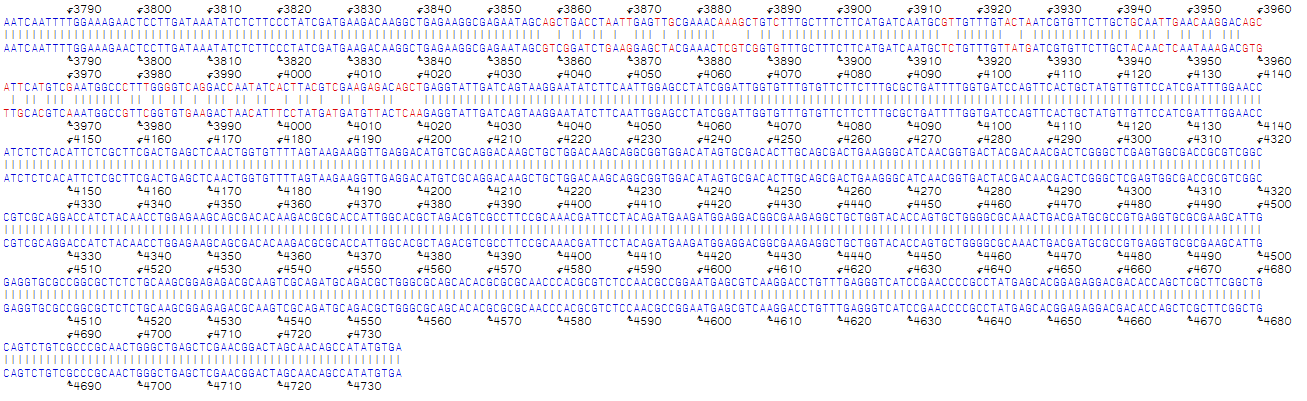


**Fig. S2. The expression pattern of TREs in different developmental stages.**


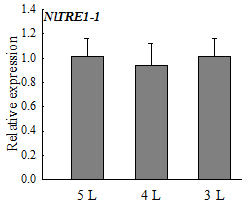

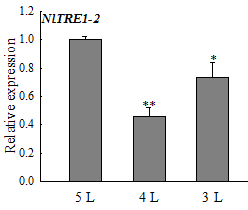

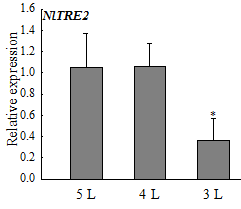


**Supplementary tables**

**Table S1. Composition of raw reads in the double stranded (ds) trehalase (TRE)1-1, dsTRE1-2, dsTRE2, and dsGFP cDNA libraries.**

| **Reads species** | **dsTRE1-1** | **dsTRE1-2** | **dsTRE2** | **dsGFP** |
| --- | --- | --- | --- | --- |
| Containing adaptor | 59724 | 63969 | 74553 | 59519 |
| Containing N | 1200 | 1330 | 1232 | 1251 |
| Low quality | 10974 | 10447 | 10047 | 9999 |
| Clean reads | 12392297 | 12002051 | 11717857 | 11883210 |
| Total reads | 12464195 | 12077797 | 11803689 | 11953979 |

GFP, green fluorescence protein used as control.

**Table S2. Comparison results against the reference genes and reference genome of *Nilaparvata lugens.***

|  | **Sample ID** | **Total Rads** | **Total Base Pairs** | **Total Mapped Reads** | **Perfect Match** | **<=2bp Mismatch** | **Unique Match** | **Muti-position Match** | **Total Unmapped Reads** |
| --- | --- | --- | --- | --- | --- | --- | --- | --- | --- |
| Mapping to reference genes | TRE1-1 | 12392297 | 607222553 | 4741160(38.26%) | 3251384 | 1489776 | 4107840(33.15%) | 633320(5.11%) | 7651137(54.91%) |
|  | TRE1-2 | 12002051 | 588100499 | 4459814(37.16%) | 3047882 | 1411932 | 3862194(32.18%) | 597620(4.98%) | 7542237(62.84%) |
|  | TRE2 | 11717857 | 574174993 | 4180602(35.68%) | 2847065 | 1333537 | 3599258(30.72%) | 581344(4.96%) | 7537255(64.32%) |
|  | GFP | 11883210 | 582277290 | 4256848(35.82%) | 2884271 | 1372577 | 3618777(30.45%) | 638071(5.37%) | 7626362(64.18%) |
| Mapping to reference genome | TRE1-1 | 12392297 | 607222553 | 9288949(74.96%) | 6051550 | 3237399 | 6805224(54.91%) | 2483725(20.04%) | 3103348(25.04%) |
|  | TRE1-2 | 12002051 | 588100499 | 8948671(74.56%) | 5820407 | 3128264 | 6551100(54.58%) | 2397571(19.98%) | 3053380(25.44%) |
|  | TRE2 | 11717857 | 574174993 | 8829104(75.35%) | 5773054 | 3056050 | 6334920(54.06%) | 2494184(19.98%) | 2888753(25.44%) |
|  | GFP | 11883210 | 582277290 | 9079399(76.41%) | 5932561 | 3146838(26.48%) | 6471401(54.46%) | 2607998(21.95%) | 2803811(23.59%) |

**Table S3. The top 10 most significantly enriched pathways.**

| **Pathway** | **DEGs with pathway annotation** | **Qvalue** | **Pathway ID** |
| --- | --- | --- | --- |
| **GFP-VS-TRE1-1** | | | |
| Metabolic pathways | 107 (18.04%) | 1.97E-06 | ko01100 |
| Amoebiasis | 47 (7.93%) | 3.32E-11 | ko05146 |
| Vibrio cholerae infection | 33 (5.56%) | 1.18E-05 | ko05110 |
| Protein digestion and absorption | 29 (4.89%) | 7.27E-09 | ko04974 |
| Pancreatic secretion | 27 (4.55%) | 1.27E-08 | ko04972 |
| Influenza A | 27 (4.55%) | 2.76E-05 | ko05164 |
| Focal adhesion | 27 (4.55%) | 6.75E-01 | ko04510 |
| MAPK signaling pathway | 25 (4.22%) | 1.74E-03 | ko04010 |
| Glycerophospholipid metabolism | 23 (3.88%) | 8.49E-08 | ko00564 |
| Neuroactive ligand-receptor interaction | 23 (3.88%) | 5.44E-03 | ko04080 |
| **GFP-VS-TRE1-2** | | | |
| Metabolic pathways | 227 (16.52%) | 1.39E-09 | ko01100 |
| Amoebiasis | 76 (5.53%) | 1.49E-10 | ko05146 |
| Regulation of actin cytoskeleton | 74 (5.39%) | 3.98E-01 | ko04810 |
| Vibrio cholerae infection | 67 (4.88%) | 4.51E-09 | ko05110 |
| Vascular smooth muscle contraction | 55 (4%) | 1.34E-01 | ko04270 |
| RNA transport | 55 (4%) | 3.87E-01 | ko03013 |
| Focal adhesion | 55 (4%) | 7.50E-01 | ko04510 |
| Purine metabolism | 44 (3.2%) | 3.49E-02 | ko00230 |
| Influenza A | 43 (3.13%) | 5.06E-04 | ko05164 |
| Epstein-Barr virus infection | 41 (2.98%) | 3.04E-02 | ko05169 |
| **GFP-VS-TRE2** | | | |
| Metabolic pathways | 53 (17.15%) | 1.18E-02 | ko01100 |
| Complement and coagulation cascades | 21 (6.8%) | 6.57E-19 | ko04610 |
| Amoebiasis | 21 (6.8%) | 5.28E-04 | ko05146 |
| Focal adhesion | 19 (6.15%) | 2.24E-01 | ko04510 |
| Regulation of actin cytoskeleton | 19 (6.15%) | 4.47E-01 | ko04810 |
| Protein digestion and absorption | 17 (5.5%) | 1.65E-05 | ko04974 |
| Pancreatic secretion | 15 (4.85%) | 8.14E-05 | ko04972 |
| RNA transport | 15 (4.85%) | 4.05E-01 | ko03013 |
| Influenza A | 14 (4.53%) | 1.19E-02 | ko05164 |
| Neuroactive ligand-receptor interaction | 14 (4.53%) | 2.38E-02 | ko04080 |
